# Targeting type Iγ phosphatidylinositol phosphate kinase overcomes oxaliplatin resistance in colorectal cancer

**DOI:** 10.7150/thno.69863

**Published:** 2022-05-20

**Authors:** Minhao Yu, Hao Wang, Wei Zhao, Xiaoxiao Ge, Wei Huang, Fengjuan Lin, Wenbo Tang, Ang Li, Sailiang Liu, Rong-Kun Li, Shu-Heng Jiang, Junli Xue

**Affiliations:** 1 Department of Gastrointestinal Surgery, Ren Ji Hospital, School of Medicine, Shanghai Jiao Tong University, Shanghai 200217, P.R. China; 2 Department of Oncology, Shanghai East Hospital, School of Medicine, Tongji University, Shanghai 200123, P.R. China; 3 Department of General Surgery, Ren Ji Hospital, School of Medicine, Shanghai Jiao Tong University, Shanghai 200217, P.R. China; 4 Institute of Oncology, Affiliated Hospital of Jiangsu University, Zhenjiang 212001, P.R. China; 5 State Key Laboratory of Oncogenes and Related Genes, Shanghai Cancer Institute, Ren Ji Hospital, School of Medicine, Shanghai Jiao Tong University, Shanghai 200240, P.R. China

**Keywords:** Chemotherapy resistance, PIP5K1C, Phosphatidylinositol kinase, Exosome, DNA damage, CD274

## Abstract

**Rationale:** Oxaliplatin is a widely used chemotherapy drug for advanced colorectal cancer (CRC) and its resistance is a major challenge for disease treatment. However, the molecular mechanism underlying oxaliplatin resistance remains largely elusive.

**Methods:** An integrative analysis was performed to determine differentially expressed genes involved in oxaliplatin resistance. Loss- and gain-of-function studies were employed to investigate the roles of type Iγ phosphatidylinositol phosphate kinase (PIPKIγ) on oxaliplatin resistance in CRC cells. Exosomes derived from CRC cell lines were assessed for PD-L1 level and the ability to promote oxaliplatin resistance. Quantitative real-time PCR, immunofluorescence, luciferase reporter assay, Western blotting and other techniques were conducted to decipher the molecular mechanism.

**Results:** PIPKIγ was identified as a critical gene related to oxaliplatin resistance in CRC. Genetic manipulation studies revealed that PIPKIγ profoundly facilitated oxaliplatin resistance and affected the expression of DNA damage repair proteins. Mechanistically, PIPKIγ promoted the expression of the immune checkpoint molecule PD-L1 via activation of NF-κB signaling pathway. Genetic silencing of PD-L1 did not affect CRC cell proliferation but significantly sensitized CRC cells to oxaliplatin. Notably, PD-L1 was revealed to be encapsulated in the exosomes, and the addition of exosomal PD-L1 to sh-PD-L1 CRC cells restored oxaliplatin resistance. Pharmacological hijacking PIPKIγ-exosomal PD-L1 axis largely reduced oxaliplatin resistance in CRC cells. *In vivo* experiments showed that PD-L1 loss significantly blocked oxaliplatin resistance and the addition of PD-L1-enriched exosomes promoted tumor growth and reduced mouse survival time.

**Conclusion:** Our findings reveal a previous unprecedented role of PIPKIγ in oxaliplatin resistance and provide a key mechanism of exosomal PD-L1 in CRC with potential therapeutics.

## Background

Colorectal cancer (CRC) is one of the most commonly diagnosed cancers with a high cancer-related mortality worldwide 1. Chemotherapy based on DNA damage agents such as oxaliplatin is still the main treatment for stage III-IV colorectal cancer. However, chemotherapy for CRC patients faces the problem of insensitivity to primary tumor chemotherapy and secondary drug resistance 2. To this end, finding new targets, increasing the sensitivity of tumor chemotherapy, and exploring new treatment strategies are of great significance for improving the survival and prognosis of CRC patients 3.

PIPKIs are a group of enzymes that synthesize phosphatidylinositol 4,5 bisphosphate (PI(4,5)P2) 4. According to the different C-terminal sequence, PIPKIs can be divided into PIPKIα, PIPKIβ, and PIPKIγ, among which PIPKIγ is the main subtype of synthetic PI(4,5)P2. PI(4,5)P2 can be used as a substrate to participate in a series of cell activities, including focal adhesion assembly, ciliogenesis, actin polymerization, vesicular trafficking, cell cycle progression, signaling pathway transduction, and gene expression 5-9. Previously, we have demonstrated that PIPKIγ plays a variety of roles in tumors, including regulation of the Warburg effect of colon cancer 10, recruiting tumor-related macrophages via regulation of CCL2 expression 11, and regulating tumor cell PD-L1 expression in CRC cells 12.

Oxaliplatin is a platinum-based chemotherapy drug widely used for CRC patients 13. Understanding the molecular mechanism of oxaliplatin resistance in CRC is of great importance to optimize current therapeutic strategies 14. In this work, we performed a screen analysis using RNA sequencing profiles of CRC patients with oxaliplatin treatment. A repertoire of differentially expressed genes (DEGs) related to oxaliplatin resistance was revealed. Subsequently, functional cell experiments and *in vivo* animal model verification were conducted to investigate the roles of PIPKIγ in oxaliplatin resistance.

## Materials and Methods

### Bioinformatic analysis

The RNA sequencing level 3 data (mRNA, miRNA, lncRNA raw counts) for colon cancer and rectal cancer were downloaded using TCGA biolinks. The data were annotated to a reference transcript set of human GRCh38/hg38 gene standard track. Differential expression analysis of individual genes was carried out using the edgeR Bioconductor package (http://bioconductor.org/packages/edgeR/). The RNAseq count data were filtered with a minimum counts-per-million (CPM) threshold present in at least 50% of samples to remove genes with low expression. Raw counts were extracted for these samples and edgeR was employed to find the differentially expressed genes (DEGs) between the two groups. The absolute log_2_[fold change (FC)] >=1 and *P* value less than 0.05 were set as a restricted condition to identify DEGs.

### Cell culture and reagents

CRC cell lines including HCT116, SW620, LoVo, and SW480 were all purchased from the American Tissue Culture Collection (ATCC, Manassas, VA, USA). All CRC cells were cultured in DMEM (Dulbecco's modified Eagle's medium, Gibco) or RPMI 1640 medium (Gibco, USA) medium supplemented with 10% (v/v) fetal bovine serum (FBS, Gibco, USA), and the antibiotics penicillin (500 units/mL) and streptomycin (200 μg/mL). All cell lines used in this study were routinely tested for mycoplasma contamination and found to be negative. CRC cells were maintained at a humidified incubator with 5% CO_2_ atmosphere. Oxaliplatin (S1224), Bay11-7082 (S2913), GW4869 (S7609), and phorbol 12-myristate 13-acetate (PMA, S7791) were purchased from Selleck (Shanghai, China).

### Generation of stable PIPKIγ and PD-L1 knockdown cells

CRC cells stably expressing sh-PIPKIγ were generated as reported previously 10. Lentiviral shRNA of negative control (sh-Ctrl) and shRNA oligonucleotides targeting PD-L1 (sh-PD-L1) were designed and synthesized by Genepharma (Shanghai, China). The lenti-virius plasmids were transfected into 293T cells using Lipofectamine 2000 (Invitrogen, USA) according to the manufacturer's protocol. Then, SW480 and LoVo cells were infected with lentivirus from the packaging cells 48 h after transfection in the presence of 5 μg/mL polybrene. After infection overnight, virus-containing medium was replaced by a new culture medium. The positive clones expressing sh-Ctrl or sh-PD-L1 were selected with 2 μg/mL puromycin for 2 weeks. The knockdown efficiency of PD-L1 was further verified by Western blotting analysis.

### Cell transfection

For overexpression of PIPKIγ, pcDNA3.1-PIPKIγ was synthesized by Genepharma (Shanghai, China). For transient expression of exogenous proteins, 3-5 × 10^5^ cells were plated in each well of 6-well plates and transfection was performed with XtremeGENE 9 (Roche, USA) following the manufacturer's instructions. Constitutively active IKKβ (CA-IKKβ) plasmid was generated as reported previously 3. The pcDNA3HA-PKB T308D/S473D plasmid, named constitutively active AKT, was generated by Genepharma (Shanghai, China).

### Western blotting

Co-Immunoprecipitation (Co-IP) experiment was performed as previously reported 2. Cell lysates were separated using 6-12% sodium dodecyl sulfate-polyacrylamide gel electrophoresis (SDS-PAGE) and then electrophoretically transferred onto PVDF membranes. After blocking with 5% (m/v) defatted milk for 1 h at room temperature, the PVDF membranes were incubated with primary antibodies at 4 °C overnight, followed by incubation with HRP-conjugated secondary antibodies at room temperature for 1 h. β-actin antibody was used as a loading control. Immunoblots were developed using the Pierce™ Western ECL Blotting substrate (ThermoFisher Scientific, Waltham, MA) and ChemiDoc Touch image system (Bio-Rad). The antibodies used in this study were listed as follows: PIPKIγ (1:2,000, Abcam, ab109192), PD-L1 (1:1,000, Cell Signaling Technology, #13684), TSG101 (1:2,000, Abcam, ab125011), CD63 (1:2,000, Abcam, ab134045), BRCA1 (1:1,000, Cell Signaling Technology, #14823), NBS1 (1:1,000, Cell Signaling Technology, #3002), RAD50 (1:2,000, Abcam, ab124682), MRE11 (1:1,000, Abcam, ab214), p-P65 (1:1,000, Cell Signaling Technology, #3033), P65 (1:1,000, Cell Signaling Technology, #4764), p-AKT (1:1,000, Cell Signaling Technology, #4060), AKT (1:1,000, Cell Signaling Technology, #4685), Cyclin D1 (1:1,000, ProteinTech, 60186-1-Ig), CDK4 (1:3,000, Abcam, ab108357), CDK6 (1:1,000, ProteinTech, 14052-1-AP), and β-actin (1:1000, Abcam, ab8227).

### Exosome purification and identification

Exosomes were isolated from CRC cell conditioned medium (CM) by differential centrifugation as reported previously 15. Briefly, CM was collected and centrifuged at 300 ×g for 30 min, 3,000 ×g for 30 min, 20,000 ×g for 30 min, and 100,000 ×g for 80 min at 4 °C. The pellets were washed twice with 1× PBS and purified by centrifugation at 100,000×g for 80 min at 4 °C. The purified exosomes were resuspended in PBS for functional experiments or used for protein detection. The protein level in the exosomes was detected by a BCA Protein Assay Kit (Thermo Fisher Scientific, USA). The size and quality of exosomes were detected by a LM10-HS NanoSlight instrument and nanoparticle tracking analysis (NTA) software.

### Plate colony formation assay

CRC cells were seeded in 6-well plate at a density of 1 × 10^3^ cells per well. After culture for 12-14 days, colonies formed were fixed with 4% paraformaldehyde, stained with 0.1% crystal violet, followed by counting under a microscope. Each experiment was performed in triplicate and repeated twice.

### Cell cycle analysis

CRC cells (3-5 × 10^5^ cells) upon indicated genetic manipulation were seeded in 6-well plates and treated with different concentrations of Oxaliplatin (0, 5, and 10 µM) for 48 h. Cells were then collected and fixed with 75% pre-cooled ethanol at -20°C overnight. Subsequently, after washing with 1× PBS three times, CRC cells were subjected to incubation with 500 μL reaction buffer containing 5 μL of RNase and 20 µL of PI (BD Biosciences, USA) at room temperature for 30 min in the dark. Finally, cell cycle distribution was analyzed with flow cytometry (BD Biosciences, USA).

### Cell apoptosis analysis

The Apo-ONE homogeneous Caspase-3/7 (protease activity) assay kit (Promega, Madison, WI, USA) was used to determine cell apoptosis. After indicated treatment for 48 h, cells were lysated at room temperature for 2 h, and Caspase 3/7 activity was detected after 2 h at an excitation wavelength of 485 nm and an emission of 528 nm using a Multi-mode microplate reader (Bio Tek Instruments, Inc., Winooski, VT, USA).

### Cell immunofluorescence assay

SW480 and LoVo cells were grown on coverslips in 24-well plates. Cells after indicated treatment, the coverslips were fixed with 4% paraformaldehyde for 30 min at room temperature, followed by permeabilized in 0.5% Triton X-100 for 3 min. Then, SW480 or LoVo were blocked with 10% (m/v) bovine serum albumin (BSA) and then incubated with anti-γ-H2AX (ab26350; Abcam). After incubating with a secondary antibody (Cell Signaling Technology, USA) for 1 h at room temperature, the cell slides were counterstained with DAPI and visualized for immunofluorescence with a laser scanning microscope (Zeiss, Germany).

### Luciferase reporter assay

CRC cells with indicated treatment were seeded at 24-well plates and allowed to grow at 60-70% confluence before transfection with 1 μg total DNA using FuGENE 6 transfection reagent (Roche, USA). The pGMNF-κB-Luc plasmids (Genomeditech, Shanghai, China) were transfected into cells according to the manufacturer's instructions. Renilla luciferase was co-transfected to normalize for transfection efficiency. After transfection for 48 h, CRC cells were collected and the luciferase activity was analyzed by a Dual Luciferase Reporter Assay System (E1910, Promega, USA).

### Tumor xenograft experiment

Pathogen-free female athymic nude mice (6-8 weeks old, 20-25 g) used in this study were maintained at SPF Laboratory Animal Center in full accordance with the guidelines by the National Institutes of Health Guide for the Care and Use of Laboratory Animals. SW480 cells (1 × 10^6^ in 100 μl) were injected subcutaneously into the right scapular region of nude mice (n = 5 per group). At the endpoint of the experiment, mice were sacrificed, and tumors were isolated, weighed, and fixed in formalin and embedded in paraffin or directly stored at -80 °C. For the drug resistance experiment, mice were randomly divided into two groups when bore visible tumors; in the test group, mice were intraperitoneally injected with 5 mg/kg oxaliplatin three times a week; in the control group, mice were treated with saline containing 0.01% DMSO; in the sh-PD-L1 + exosomes group, mice were injected with 100 μl of PD-L1 WT exosomes resuspended in PBS via the tail vein, three times a week. All the animal experiments were approved by the Animal Care and Use Committee of Shanghai East Hospital, Tongji University School of Medicine. All experiments strictly followed the guidelines for the investigation of experimental pain in conscious animals to minimize animal suffering and improve animal welfare.

### Statistical analysis

All data were presented in the form of means ± standard deviation (SD). GraphPad Prism (GraphPad Software, Inc., San Diego, CA, USA) software was used for statistical analyses. The Student paired t-test was used to estimate statistically significant differences between the two groups. One-way analysis of variance (ANOVA) followed by two-tailed post hoc Tukey's multiple comparison test was used for multiple comparisons. A two-sided p-value less than 0.05 was considered statistically significant. Survival rates were calculated using the Kaplan-Meier method and comparisons were performed using the Log-rank test. *P < 0.05, **P < 0.01 and ***P < 0.001.

## Results

### Identification of PIPKIγ as a critical gene involved in oxaliplatin resistance of CRC

**Figure 1:**
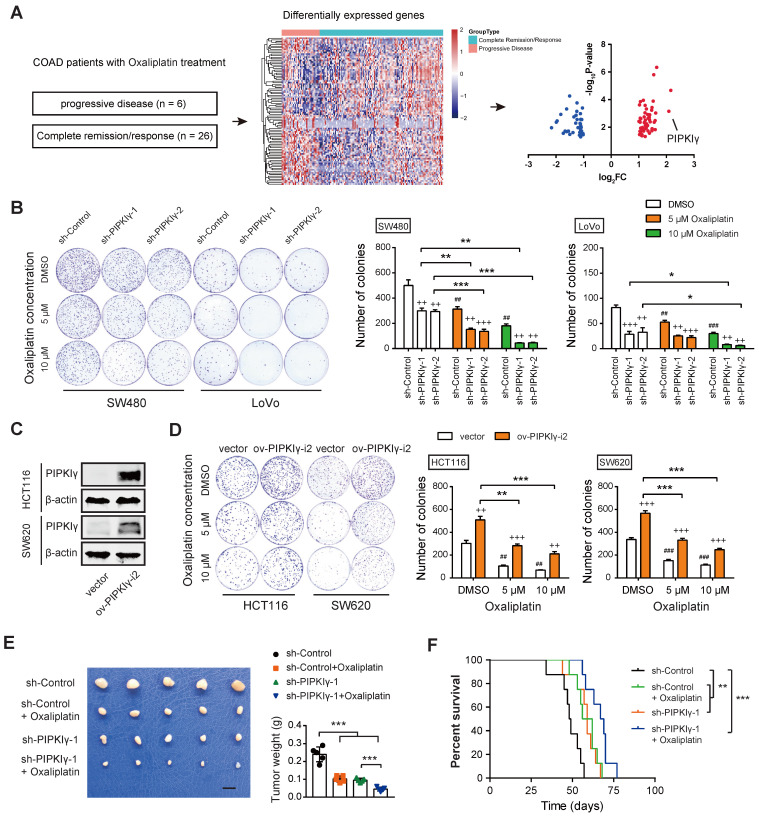
** Identification of PIPKIγ as a critical gene involved in oxaliplatin resistance of CRC.** (**A**) Screen model for differentially expressed genes related to oxaliplatin resistance of CRC. (**B**) The effects of oxaliplatin treatment on sh-Ctrl and sh-PIPKIγ SW480 and LoVo cell proliferation were measured by plate colony formation assay. (**C**) The overexpression efficiency of PIPKIγ in HCT116 and SW620 cells was verified by Western blotting analysis. (**D**) The effects of oxaliplatin treatment on ov-vector and ov-PIPKIγ-i2 HCT116 and SW620 cell proliferation were measured by plate colony formation assay. (**E**) sh-Ctrl, and sh-PIPKIγ-1 SW480 cells were injected subcutaneously into the left forelimb of nude mice (n = 5 per group); after bearing palpable tumors, animals were treated with oxaliplatin; four weeks later, mice were sacrificed and tumor weights in each group were shown; scale bar: 1 cm. (**F**) Kaplan-Meier analyses of overall survival in mice from indicated subcutaneous xenograft. + indicates comparisons between sh-PIPKIγ and sh-Ctrl or comparisons between ov-PIPKIγ-i2 and ov-vector, ^++^P < 0.01, ^+++^P < 0.001; # represents comparison between DMSO and oxaliplatin treatment, ^##^P < 0.01, ^###^P < 0.001; asterisks represent indicated comparisons, *P < 0.05, **P < 0.01, ***P < 0.001. P values are derived from the ANOVA followed by post hoc Tukey's multiple comparison test (**B**, **D**, **E**) or log-rank test (**F**).

To identify the key player implicated in oxaliplatin resistance, we mined the RNAseq data of CRC patients with oxaliplatin treatment in the TCGA cohort. As a result, 6 patients with clinical progressive disease and 26 patients with a complete response were subjected to identify DEGs related to oxaliplatin resistance. Among the DEGs, PIPKIγ and C6orf15 had a log_2_FC larger than 2 (**Figure 1A** and **Table S1**). In this study, we focused on the investigation of the link between PIPKIγ and oxaliplatin resistance. To determine the cellular functions of PIPKIγ, two cell lines (SW480 and LoVo) with higher PIPKIγ protein levels were used for loss-of-function studies; in contrast, two cell lines (HCT116 and SW620) with lower PIPKIγ protein levels were used for gain-of-function studies 10. As reported previously 10, knockdown of PIPKIγ inhibited CRC cell proliferation (**Figure 1B**). When oxaliplatin was added, cell proliferation of SW480 and LoVo was further suppressed, suggesting a connection between PIPKIγ and oxaliplatin resistance (**Figure 1B**). To further confirm this finding, we overexpressed PIPKIγ-i2 in HCT116 and SW620 cells (**Figure 1C**). Indeed, PIPKIγ-i2 overexpression largely compromised oxaliplatin resistance in HCT116 and SW620 cells, especially at a concentration of 10 μM oxaliplatin (**Figure 1D**). Consistently, data from cell cycle and cell apoptosis analysis also supported the PIPKIγ-dependent oxaliplatin resistance (**Figure S1**). Moreover, we generated a subcutaneous xenograft model to investigate the PIPKIγ-dependent oxaliplatin resistance *in vivo*. The results showed that oxaliplatin significantly inhibited CRC tumor growth, and the inhibitory effect can be amplified by the knockdown of PIPKIγ (**Figure 1E**). Moreover, the beneficial effect of oxaliplatin on mouse survival was further improved by the knockdown of PIPKIγ (**Figure 1F**).

Oxaliplatin blocks DNA replication, transcription, and repair through cross-linking with DNA double-strands 16. The MRN complex is an important complex for detecting and repairing double-stranded DNA damage and includes MRE11, NBS1, and RAD50 17. The three proteins of the MRN complex are usually located in the cytoplasm, and enter the nucleus from the cytoplasm to play a role after the cell DNA double-strand damage occurs. When the expression of MRN complex is low, CRC cells are sensitive to oxaliplatin chemotherapy, and vice versa. One of the important roles of BRCA1 in tumors is to promote DNA homologous recombination and accelerate DNA damage repair 18. MRN complex and BRCA1 are the main proteins in response to DNA damage, and both regulate the sensitivity of cells to oxaliplatin. Western blotting analysis showed that the expression of BRCA1, NBS1, RAD50, and MRE11 was remarkably down-regulated in PIPKIγ-silenced cells compared with the control cells. Among them, BRCA1 was down-regulated most significantly, suggesting that DNA damage response is reduced (**Figure S2A**). However, Co-IP experiment showed that PIPKIγ did not interact with BRCA1 directly in colorectal cancer cells (**Figure S2B**). Collectively, PIPKIγ may affect the sensitivity of CRC cells to oxaliplatin by regulating DNA damage response.

### PD-L1 is regulated by PIPKIγ and contributes to oxaliplatin resistance in CRC

**Figure 2:**
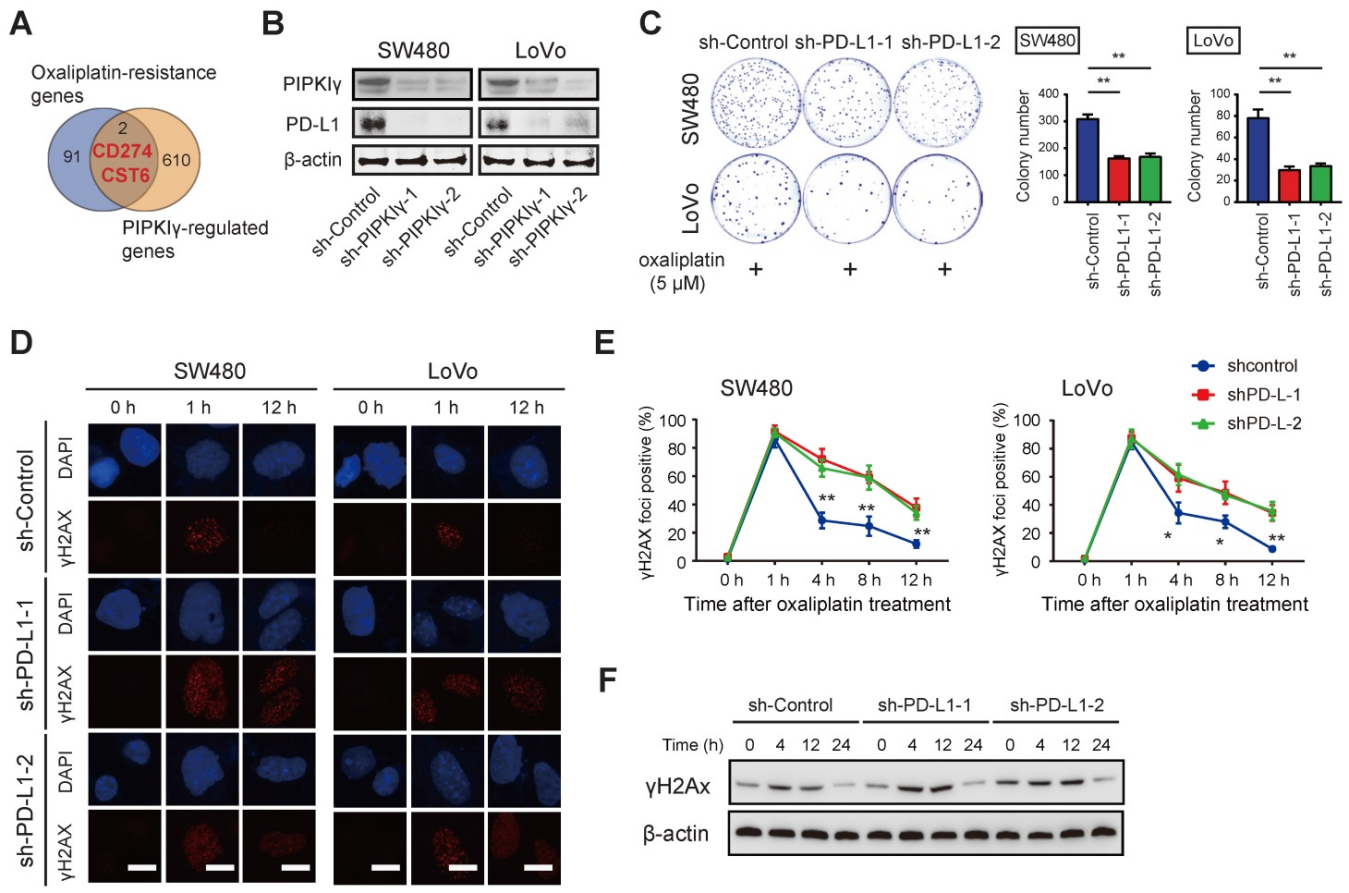
** PD-L1 is a target gene of PIPKIγ in CRC.** (**A**) The Venn diagram showed that 612 differentially expressed genes (DEGs) obtained from transcriptome sequencing analysis were combined with oxaliplatin resistance-related genes, and two DEGs (PD-L1 and CST6) were found. (**B**) Cell lysates of sh-Ctrl and sh-PIPKIγ SW480 and LoVo cells were collected for immunoblotting analyses using PIPKIγ and PD-L1 antibodies; β-actin was loaded as a control. (**C**) The effects of oxaliplatin treatment (5 μM) on sh-Ctrl and sh-PD-L1 SW480 and LoVo cell proliferation were measured by plate colony formation assay. (**D**) Immunofluorescence staining showed the change of DNA damage index γH2AX after oxaliplatin intervention in sh-Ctrl and sh-PD-L1 SW480 and LoVo cells. (**E**) H2AX index at indicated time points after oxaliplatin intervention in sh-Ctrl and sh-PD-L1 SW480 and LoVo cells. (**F**) Western blotting analysis of γH2AX after oxaliplatin intervention in sh-Ctrl and sh-PD-L1 SW480 cells. *P < 0.05, **P < 0.01. P values are derived from the ANOVA followed by post hoc Tukey's multiple comparison test.

To explore the molecular mechanism of PIPKIγ regulating DNA damage response, we conducted transcriptome sequencing of sh-Ctrl and sh-PIPKIγ-1 SW480 cells and identified 612 DEGs 10. By merging with DEGs related to oxaliplatin resistance, two DEGs, PD-L1 and CST6, were found (**Figure 2A**). Previously, we have confirmed that PIPKIγ knockdown inhibits PD-L1 mRNA transcription and reduces the expression of PD-L1 protein 12. Intriguingly, Western blotting results showed that the expression of PD-L1 protein in CRC cells was reduced by PIPKIγ knockdown (**Figure 2B**). PIPKIγ is an enzyme producing PtdIns(4,5)P2. To determine whether the kinase activity of PIPKIγ is required for its function in modulating PD-L1 and CRC proliferation or not, we overexpressed kinase-dead PIPKIγ (PIPKIγ-KD) in HCT116 and SW620 cells (with lower intrinsic PIPKIγ expression) and evaluated PD-L1 expression and CRC proliferation (**Figure S3A**). As a result, PIPKIγ-KD failed to increase PD-L1 expression and CRC proliferation (**Figure S3A-B**). PtdIns(4,5)P2 can be cleaved by phospholipase C to generate diacylglycerol that activates PKC and PKD. To investigate whether PKC or PKD is downstream of PIPKIγ for PD-L1 expression, we treated CRC cells with 10 ng/mL phorbol 12-myristate 13-acetate (PMA, PKC activator) and measured PD-L1 expression. Indeed, PMA enhanced PD-L1 expression in both HCT116 and SW620 cells (**Figure S3C**). Therefore, PtdIns(4,5)P2 likely promotes PD-L1 expression and CRC proliferation.

Next, we thoroughly studied the link between PD-L1 and oxaliplatin sensitivity in CRC. As documented previously 9, high expression of PD-L1 is related to tumor platinum resistance; the expression of PD-L1 is increased in colon cancer drug-resistant cell lines; silencing the expression of PD-L1 can increase the sensitivity of platinum-based drugs to chemotherapy. Likewise, genetic silencing of PD-L1 had no significant effect on colorectal cancer cell proliferation but significantly sensitized SW480 and LoVo cells to oxaliplatin treatment as evidenced by the diminished number of colonies (**Figure 2C** and **Figure S4A-B**). In PD-L1 knockdown cells, the baseline level of DNA damage index γH2AX was increased and the later dephosphorylation was delayed (**Figure 2D**). Upon oxaliplatin treatment, PD-L1 knockdown CRC cells showed G2/M cell cycle arrest and increased cell apoptosis as demonstrated by reduced S phase, increased Caspase3/7 activity, and expression of proteins involved in cell proliferation (Cyclin D1, CDK4, and CDK6) (**Figure S4C-E**). Collectively, these findings suggest that PIPKIγ-mediated PD-L1 expression may mediate oxaliplatin resistance in CRC cells.

### PIPKIγ regulates PD-L1 expression via activation of NF-κB

**Figure 3:**
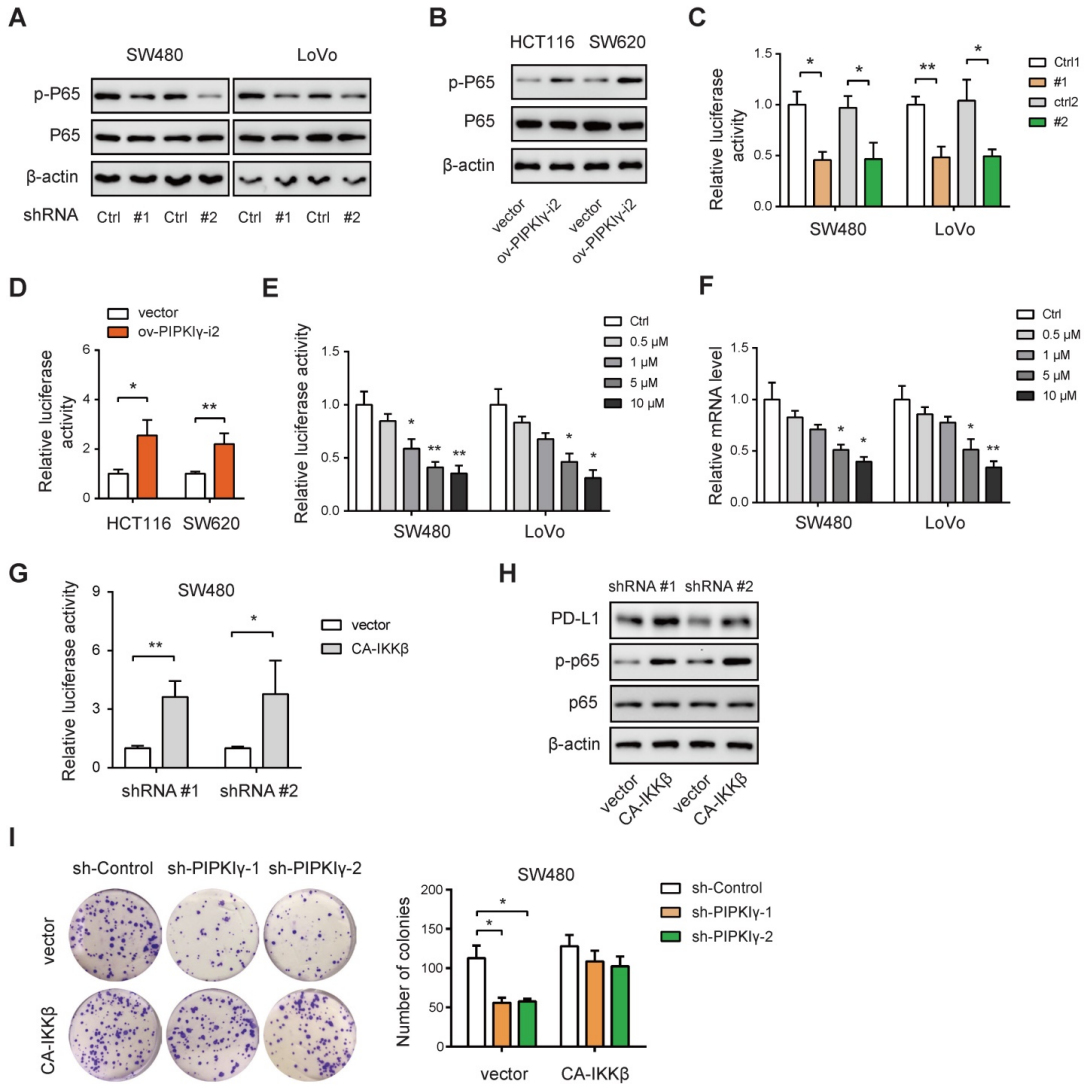
** PIPKIγ regulates PD-L1 expression via activation of NF-κB.** (**A**) The effects of PIPKIγ knockdown on the activation of NF-κB signaling pathway in SW480 and LoVo cells were analyzed by Western blotting. (**B**) The effects of PIPKIγ overexpression on the activation of NF-κB signaling pathway in HCT116 and SW620 cells were analyzed by Western blotting. (**C**) The effects of PIPKIγ knockdown on the transcriptional activity of NF-κB in SW480 and LoVo cells were determined by luciferase reporter assay. (**D**) The effects of PIPKIγ overexpression on the transcriptional activity of NF-κB in HCT116 and SW620 cells were determined by luciferase reporter assay. (**E**) The effects of NF-κB inhibitor Bay11-7082 on the transcriptional activity of NF-κB in SW480 and LoVo cells were determined by luciferase reporter assay. (**F**) The effects of Bay11-7082 on the mRNA level of PD-L1 in SW480 and LoVo cells were determined by real-time qPCR analysis. (**G**) The effects of CA-IKKβ on the NF-κB transcriptional activity in sh-PIPKIγ SW480 cells were determined by luciferase reporter assay. (**H**) Western blotting analysis of the effect of CA-IKKβ on NF-κB pathway and PD-L1 expression. (**I**) The impact of CA-IKKβ on the cell proliferation of sh-PIPKIγ SW480 cells was determined by plate colony assay. *P < 0.05; **P < 0.01. P values are derived from Student paired t-test (**C**, **D**, **G**) or the ANOVA followed by post hoc Tukey's multiple comparison test (**E**, **F**, **I**).

Next, we pursued the molecular mechanism by which PIPKIγ regulates PD-L1 expression in CRC cells. PIPKIγ can activate the NF-κB signaling pathway to induce the transcription of PD-L1 12. Indeed, knockdown of PIPKIγ significantly down-regulated the phosphorated level of P65 (p-P65) in SW480 and LoVo cells (**Figure 3A**), while overexpression of PIPKIγ increased p-P65 level in HCT116 and SW620 cells (**Figure 3B**). Consistently, PIPKIγ knockdown reduced the transcriptional activity of NF-κB as determined by luciferase reporter assay (**Figure 3C**). In contrast, PIPKIγ overexpression boosted NF-κB activity (**Figure 3D**). To evaluate the regulatory role of NF-κB in PD-L1 expression, SW480 and LoVo cells were treated with a small-molecule inhibitor of NF-κB, Bay11-7082. Expectedly, Bay11-7082 treatment led to inhibition of NF-κB activity (**Figure 3E**) and reduced the mRNA of PD-L1 in a dose-dependent manner (**Figure 3F**). Finally, ectopic expression of the constitutively active IKKβ (CA-IKKβ) in sh-PIPKIγ SW480 cells largely restored NF-κB activity (**Figure 3G**) and increased the protein level of PD-L1 (**Figure 3H**). Previously, we revealed an Akt-dependent mechanism for PIPKIγ-induced of NF-kB activation. Indeed, PIPKIγ knockdown weakened the activation of AKT in SW480 cells, while PIPKIγ overexpression increased the phosphorated level of AKT (**Figure S5A-B**). Inhibition of AKT with LY194002 blocked PIPKIγ-mediated P65 phosphorylation, NF-κB reporter activity, and PD-L1 expression (**Figure S5C-D**). In contrast, overexpression of exogenous AKT^T308D/S473D^ restored P65 phosphorylation, NF-κB reporter activity, and PD-L1 expression in shPIPKIγ SW480 cells (**Figure S5E-F**). Taken together, PIPKIγ-induced activation of NF-κB signaling is essential for PD-L1 expression in CRC.

### Exosomal PD-L1 is associated with oxaliplatin resistance of CRC cells

**Figure 4:**
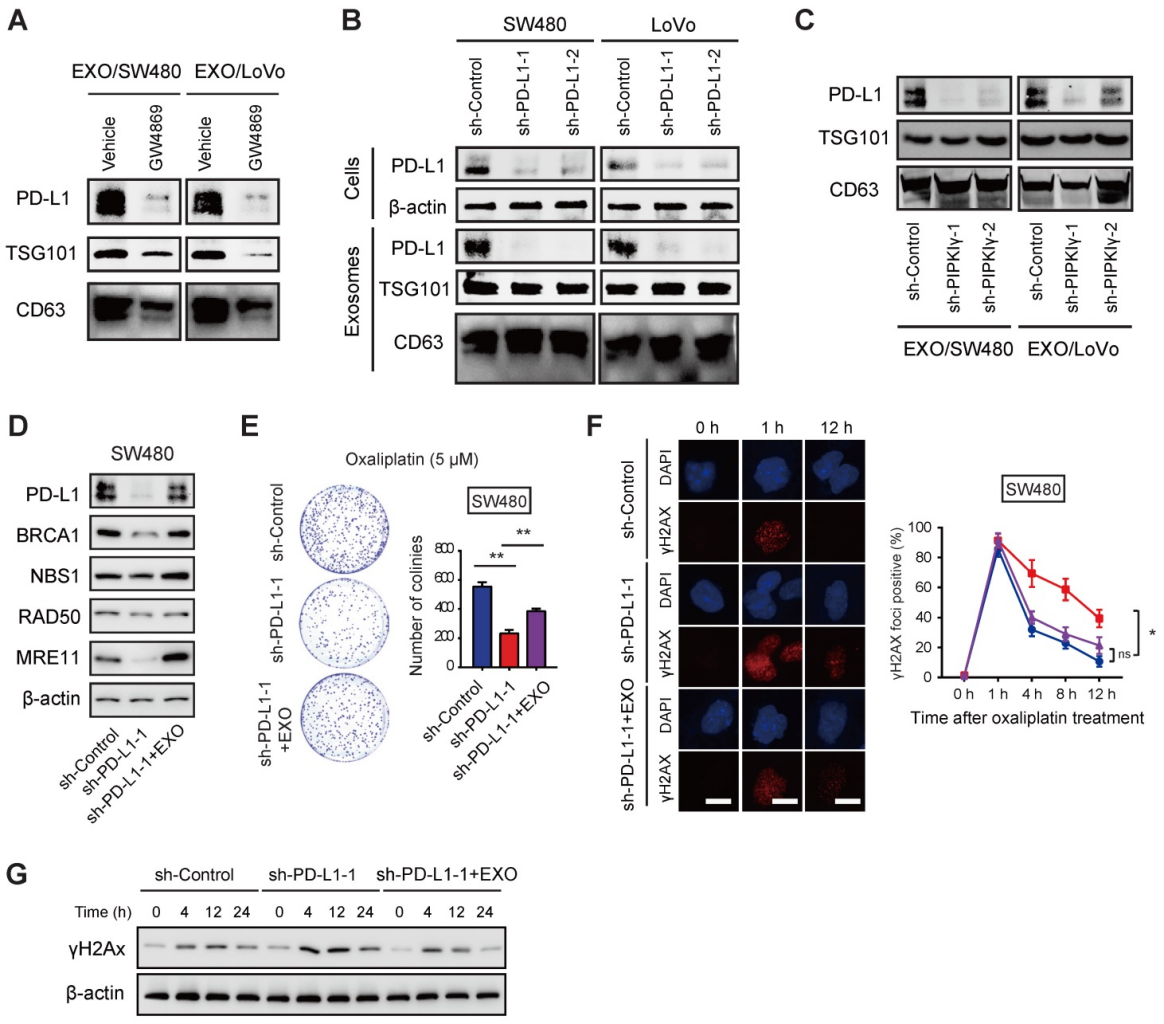
** Exosomal PD-L1 is involved in oxaliplatin resistance of CRC cells.** (**A**) SW480 and LoVo cells were cultured *in vitro* in the presence or absence of GW4869 treatment, and the exosomes were separated by ultracentrifugation. The protein expression of PD-L1 in the exosomes was determined by Western blotting analysis; CD63 and TSG101 were used as protein marker for exosomes. (**B**) The effects of PD-L1 knockdown on the cellular and exosomal PD-L1 in SW480 and LoVo cells were analyzed by Western blotting. (**C**) The effects of PIPKIγ knockdown on the exosomal PD-L1 in SW480 and LoVo cells were analyzed by Western blotting. (**D**) SW480 were cultured and divided into sh-Ctrl group and sh-PD-L1 group; the third group is sh-PD-L1 cells with treatment of PD-L1-rich exosomes, then western blotting was used to detect the expression of DNA damage repair-related proteins (BRCA1, NBS1, RAD50, and MRE11). (**E**) Comparison of cell proliferation of sh-Ctrl, sh-PD-L1, and sh-PD-L1 + exosomes groups in the presence of 5 μM oxaliplatin. (**F**) Immunofluorescence staining showed the change of DNA damage index γH2AX in sh-Ctrl, sh-PD-L1, and sh-PD-L1 + exosomes groups. (**G**) Cell lysates from cells indicated treatment and time points were subjected to immunoblotting of γH2AX. *P < 0.05, **P < 0.01; ns indicates not significant. P values are derived from the ANOVA followed by post hoc Tukey's multiple comparison test.

Exosomes are small membrane vesicles that contain a variety of biomolecules including protein, RNA, DNA, and cytokines, which reach the recipient cell through lateral transport to mediate intercellular signal transduction 19. PD-L1 can be secreted in large quantities in the form of exosomes 20. Emerging studies have shown that exosomal PD-L1 contributes to immunotherapy resistance by several mechanisms 21-23. In addition, accumulated studies have found that in different tumors, circulating exosomal PD-L1 can predict the sensitivity of immunotherapy, which is related to tumor prognosis 24, 25. Interestingly, PD-L1 was abundantly expressed in exosomes derived from colorectal cancer cells, and the nSMase inhibitor GW4869 or PD-L1 knockdown significantly reduced the expression of PD-L1 in exosomes (**Figure 4A-B**). In line with this finding, knockdown of PIPKIγ also led to a remarkable down-regulation of exosomal PD-L1 in SW480 and LoVo cells (**Figure 4C**).

Next, we tested whether exosomal PD-L1 is involved in the sensitivity of CRC cells to oxaliplatin. Previously, we have confirmed that IFN-γ stimulation can significantly increase the expression of exosomal PD-L1 in tumor cells 12. After 48 h of IFN-γ action, the SW480 cell supernatants were collected and exosomes were separated. As a result, exosomal PD-L1 protein in PD-L1 knockdown SW480 cells was significantly attenuated and can be restored by the addition of PD-L1-enriched exosomes (**Figure 4D**). Moreover, the addition of PD-L1-enriched exosomes restored the expression of BRCA1 and MRN complex in PIPKIγ KO cells in response to oxaliplatin treatment (**Figure 4D**). In line with this observation, growth inhibition induced by PD-L1 knockdown upon oxaliplatin treatment was also largely compromised by PD-L1-enriched exosomes (**Figure 4E**). Likewise, γH2AX staining and Western blotting analysis confirmed the role of exosomal PD-L1 in CRC cells upon oxaliplatin treatment (**Figure 4F-G**). Therefore, these findings above suggest that exosomal PD-L1 is largely involved in oxaliplatin resistance in CRC.

### Targeting the PIPKIγ-exosomal PD-L1 axis reverses oxaliplatin resistance in CRC cells

**Figure 5:**
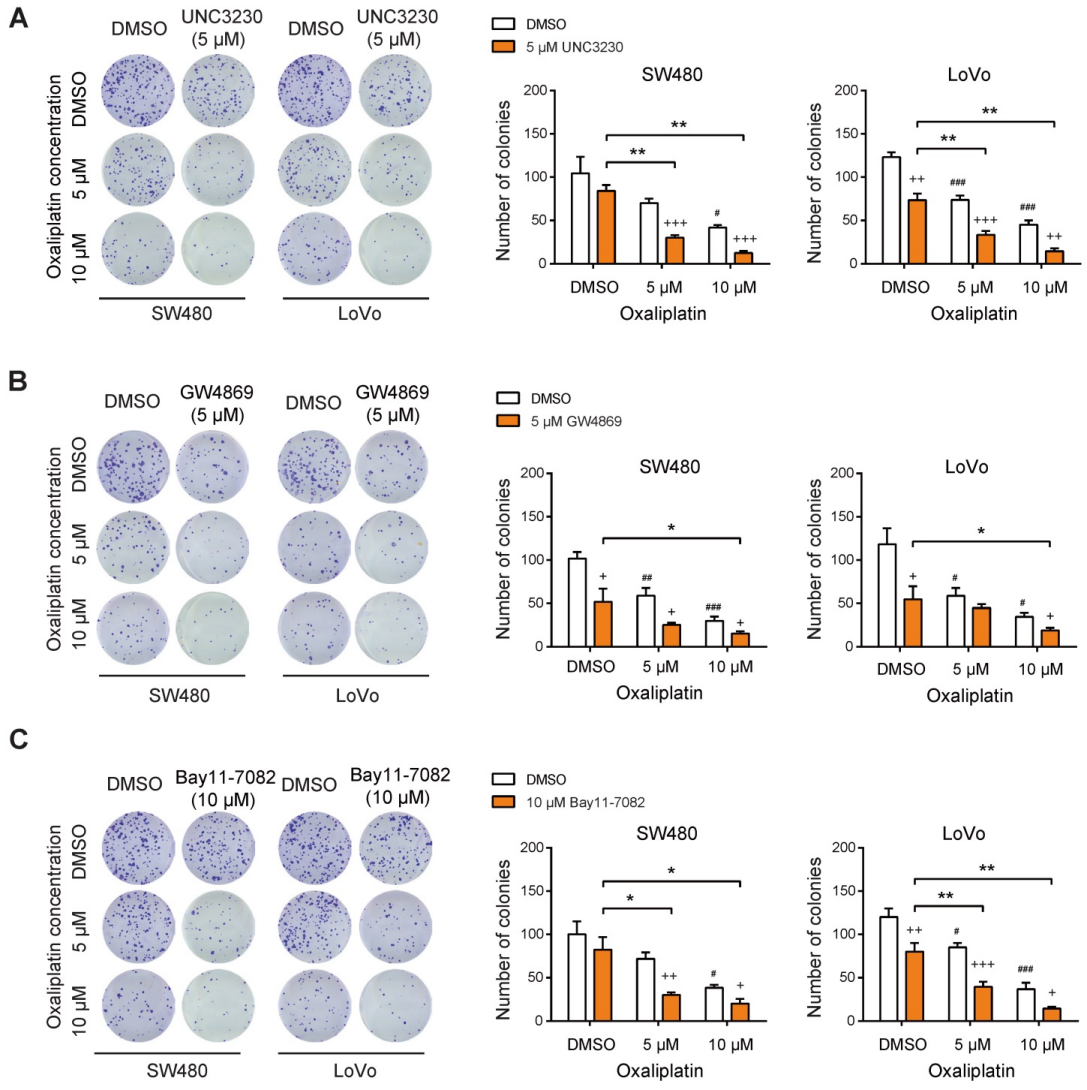
** Targeting PIPKIγ-exosomal PD-L1 axis reverses oxaliplatin resistance in CRC cells.** (**A**) The effects of oxaliplatin on SW480 and LoVo cell proliferation upon treatment with 5 μM UNC3230 were measured by plate colony formation assay. (**B**) The effects of oxaliplatin on SW480 and LoVo cell proliferation upon treatment with 5 μM GW4869 were measured by plate colony formation assay. (**C**) The effects of oxaliplatin on SW480 and LoVo cell proliferation upon treatment with 10 μM Bay11-7082 were measured by plate colony formation assay. + indicates comparison between inhibitors (UNC3230, GW4869, or Bay11-7082) and DMSO, ^+^P < 0.05, ^++^P < 0.01, ^+++^P < 0.001; # represents comparison between DMSO and oxaliplatin treatment, ^#^P < 0.05, ^##^P < 0.01, ^###^P < 0.001; asterisks represent indicated comparisons, *P < 0.05, **P < 0.01. P values are derived from the ANOVA followed by post hoc Tukey's multiple comparison test.

From the point of view of targeted therapies, we pharmacologically inhibited the PIPKIγ-exosomal PD-L1 axis with three small molecular inhibitors: 1) UNC3230, a specific inhibitor of PIPKIγ; 2) the exosome production inhibitor GW4869; 3) the NF-κB inhibitor Bay11-7082. Consistent with our previous findings 10, inhibition of PIPKIγ activity with UNC3230 led to a mild to moderate reduction in CRC cell proliferation. In combination with oxaliplatin treatment, UNC3230 remarkably suppressed SW480 and LoVo cell proliferation, further confirming the regulatory role of PIPKIγ in oxaliplatin resistance (**Figure 5A**). Likewise, both GW4869 and Bay11-7082 exerted tumor-suppressive roles and increased chemosensitivity to oxaliplatin treatment in SW480 and LoVo cells (**Figure 5B-C**) as evidenced by diminished number of colonies.

### PD-L1 knockdown reverses oxaliplatin resistance *in vivo*

**Figure 6:**
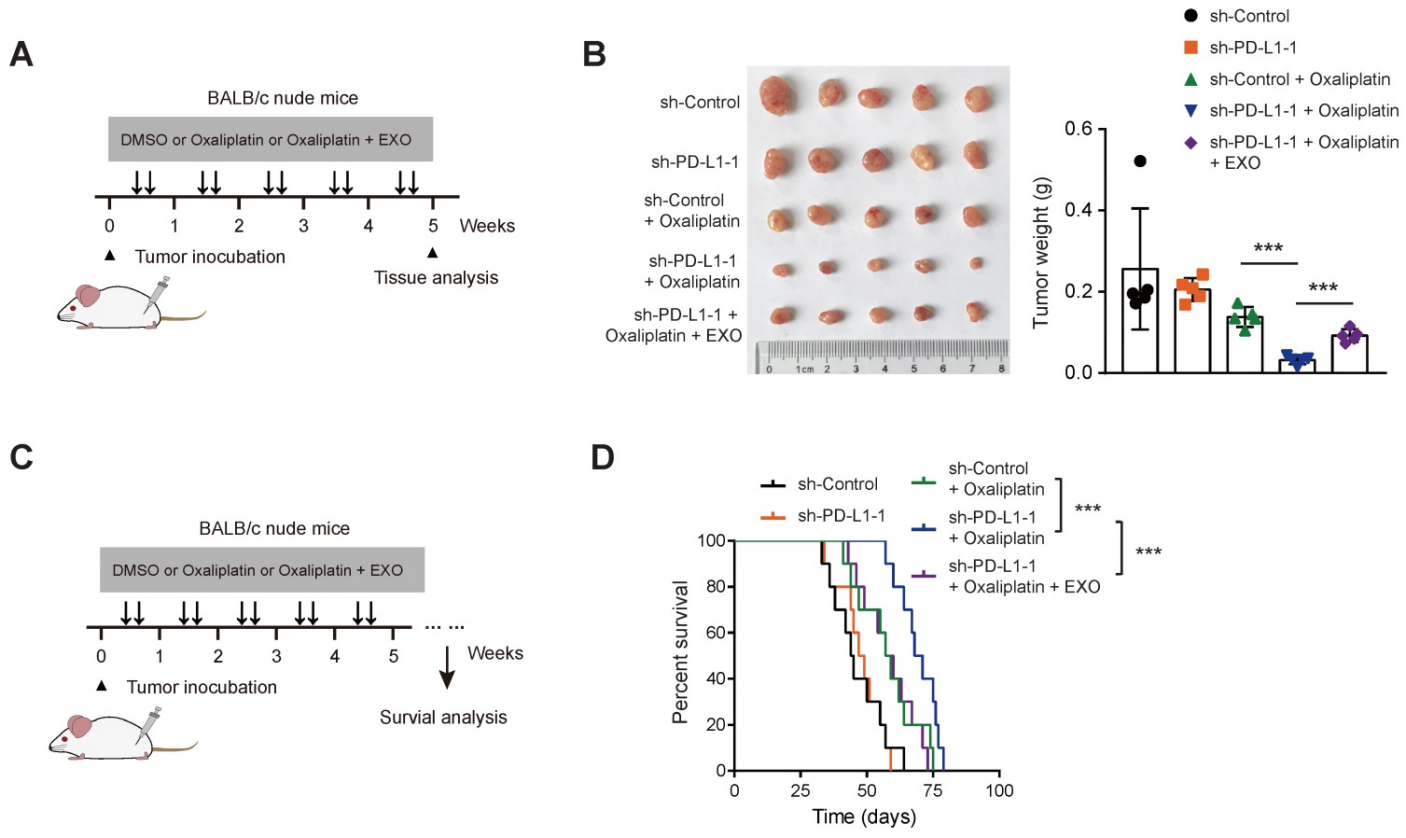
** PD-L1 knockdown reverses oxaliplatin resistance *in vivo*.** (**A**) Treatment scheme for monitoring tumor burden upon PD-L1 knockdown. (**B**) sh-Ctrl, and sh-PD-L1 SW480 cells were injected subcutaneously into the left forelimb of nude mice (n = 5 per group); after bearing palpable tumors, animals were injected with oxaliplatin or oxaliplatin/exosomes; four weeks later, mice were sacrificed and tumor weights in each group were shown. (**C**) Treatment scheme for mouse survival analysis upon PD-L1 knockdown. (**D**) Kaplan-Meier analyses of overall survival in mice from indicated subcutaneous xenograft. **P < 0.01 and ***P < 0.001. P values are derived from the ANOVA followed by post hoc Tukey's multiple comparison test (**B**) or log-rank test (**D**).

**Figure 7:**
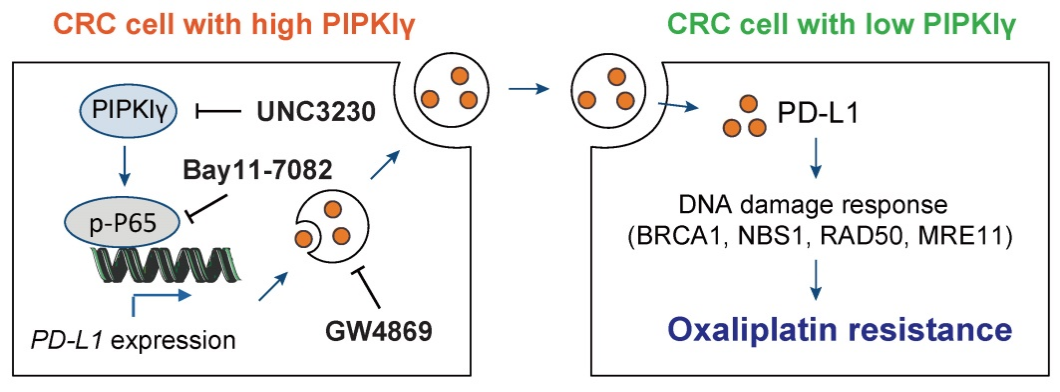
** Mechanism model for PIPKIγ-mediated oxaliplatin resistance in CRC.** PIPKIγ is frequently expressed in CRC cells with oxaliplatin resistance. Highly expressed PIPKIγ induces the activation of the NF-κB signaling pathway and subsequent transcription of PD-L1 in CRC cells. Then, PD-L1 is packaged into the exosomes and transferred to the oxaliplatin-sensitive CRC cells, which display lower expression of PIPKIγ. PD-L1 finally contributes to oxaliplatin resistance though modulation of DNA damage response. Hijacking PIPKIγ-exosomal PD-L1 axis with UNC3230, Bay11-7082, or GW4869 largely reverses oxaliplatin resistance in CRC cells.

Finally, SW480 cells stably transfected with sh-Ctrl and sh-PD-L1 were injected subcutaneously into nude mice. After bearing palpable tumors, animals were injected with oxaliplatin intraperitoneally for intervention (**Figure 6A**). Expectedly, PD-L1 knockdown did not affect *in vivo* tumor growth (**Figure 6B**). When in combination with oxaliplatin treatment, PD-L1 knockdown significantly retarded tumor growth, and the survival time was significantly prolonged; while in another group of PD-L1 knockdown, tail vein injection of PD-L1-enriched exosomes restored tumor growth, and mouse survival time was shortened (**Figure 6C-D**). Taken together, the above experiments indicate that exosomal PD-L1 is related to the sensitivity of CRC cells to oxaliplatin. Exosomes might act as a shutter to propaganda PD-L1-mediated oxaliplatin resistance, thus rendering oxaliplatin-sensitive CRC cells (with lower intrinsic PD-L1 expression) resistant to oxaliplatin treatment (**Figure 7**).

## Discussion

In this study, we for the first time uncovered the role of PIPKIγ in oxaliplatin chemotherapy resistance of CRC and deciphered its specific mechanism via regulation of exosomal PD-L1. Our findings provide several lines of evidence supporting the critical role of PIPKIγ-exosomal PD-L1 axis in oxaliplatin resistance. First, the expression of PIPKIγ was significantly increased in both CRC patients with progressive disease. Second, loss-of-function and gain-of-function studies suggested that PIPKIγ is critically involved oxaliplatin resistance. Third, PIPKIγ modulated the activity of NF-κB to increase PD-L1 expression. Fourth, exosomal PD-L1 is related to oxaliplatin resistance in CRC cells. Fifth, pharmacological blocking of the PIPKIγ-exosomal PD-L1 axis attenuated oxaliplatin resistance *in vitro* and *in vivo*.

PIPKIγ plays multiple oncogenic roles in human cancers including breast cancer metastasis 8, colorectal cancer glycolysis 10, lung cancer cell growth 26, and pancreatic cancer progression 27. Here, we reported a novel role of PIPKIγ in regulating oxaliplatin resistance. PIPKIγ can initiate a variety of signaling pathways to execute diverse cellular functions, such as PI3K-AKT signaling, AKT-STAT3 signaling, and NF-κB signaling 11, 12, 27. In this study, PD-L1 was revealed to be a direct target of PIPKIγ to mediate oxaliplatin resistance, at least to some extent. Of note, we cannot rule out other possibilities or downstream targets of PIPKIγ in driving oxaliplatin resistance of CRC cells. Apart from PIPKIγ, many other differentially expressed genes associated with oxaliplatin resistance were also revealed. Further investigations focus on these genes may also yield great findings to overcome oxaliplatin resistance in CRC.

PD-L1 is extensively documented regarding its known roles in suppressing the anti-tumor immune response 28. However, accumulating studies reveal distinct tumor-intrinsic and immune-independent functions for PD-L1 in regulating diverse malignant phenotypes, such as tumor growth, distant metastasis, epithelial-to-mesenchymal transition (EMT), stemness, and resistance to chemotherapy 29, 30. Notably, several studies documented the versatile roles of PD-L1 in regulating chemoresistance in human cancers 31, 32. For instance, PD-L1 can directly interact with integrin β6 and activate the downstream FAK signaling pathway to facilitate chemoresistance in bladder cancer 31. In head and neck squamous cell carcinoma, PD-L1 and MRN play a synergistic role in platinum-based chemoresistance 9. Consistently, PD-L1 expression was increased in cisplatin chemoresistant but not the chemosensitive cell line and PD-L1 can interact directly with the MRN complex 32. Moreover, intracellular PD-L1 has been reported to compete with the RNA exosome to modulate the DNA damage response in cancers 33. These studies clearly demonstrated that tumor cell-intrinsic PD-L1 contributes to cancer chemoresistance in multiple tumor types. In the present study, we further broadened the previous unprecedented role of PD-L1 in regulating oxaliplatin resistance of colorectal cancer. More interestingly, exosomes might act as a shutter to propaganda PD-L1-mediated oxaliplatin resistance. Indeed, blocking the production of exosomes with GW4869 reversed oxaliplatin resistance. Because the expression of BRCA1 and components of MRN complex were remarkably reduced after PIPKIγ knockdown and there was no direct physical interaction between PIPKIγ and BRCA1, it is possible that PD-L1 regulates oxaliplatin resistance via direct interaction with the MRN complex as the expression of BRCA1 and MRN complex in PIPKIγ KO cells was restored by PD-L1-enriched exosomes. Indeed, more works are encouraged to interrogate the detailed mechanism basis for PD-L1-mediated oxaliplatin resistance in CRC.

In conclusion, our findings and these studies suggest that targeting PIPKIγ-exosomal PD-L1 axis might provide a new therapeutic target for CRC patients who are not sensitive to or resistant to oxaliplatin chemotherapy. Functional investigation of PD-L1-mediated DNA damage response or other signaling network will help reveal the mechanisms that underlie the oxaliplatin resistance of CRC.

## Supplementary Material

Supplementary figures and table.

## Abbreviations

CRC: colorectal cancer
PIPKIγ: type Iγ phosphatidylinositol phosphate kinase
PI(4,5)P2: phosphatidylinositol 4,5 bisphosphate
TCGA: The Cancer Genome Atlas
DEGs: differentially expressed genes
PD-L1: programmed death-ligand 1
IFN-γ: interferon γ
