# Supplementary material for: Targeting type Iγ phosphatidylinositol phosphate kinase overcomes oxaliplatin resistance in colorectal cancer
Source: Theranostics. 2022 May 20;12(9):4386–98. doi: 10.7150/thno.69863 (PMC9169372; doi:10.7150/thno.69863)
Supplement: Supplementary file 1 — Supplementary figures and table. [file thnov12p4386s1.pdf]

Supplementary information

Supplementary Figures

Supplementary Figure 1

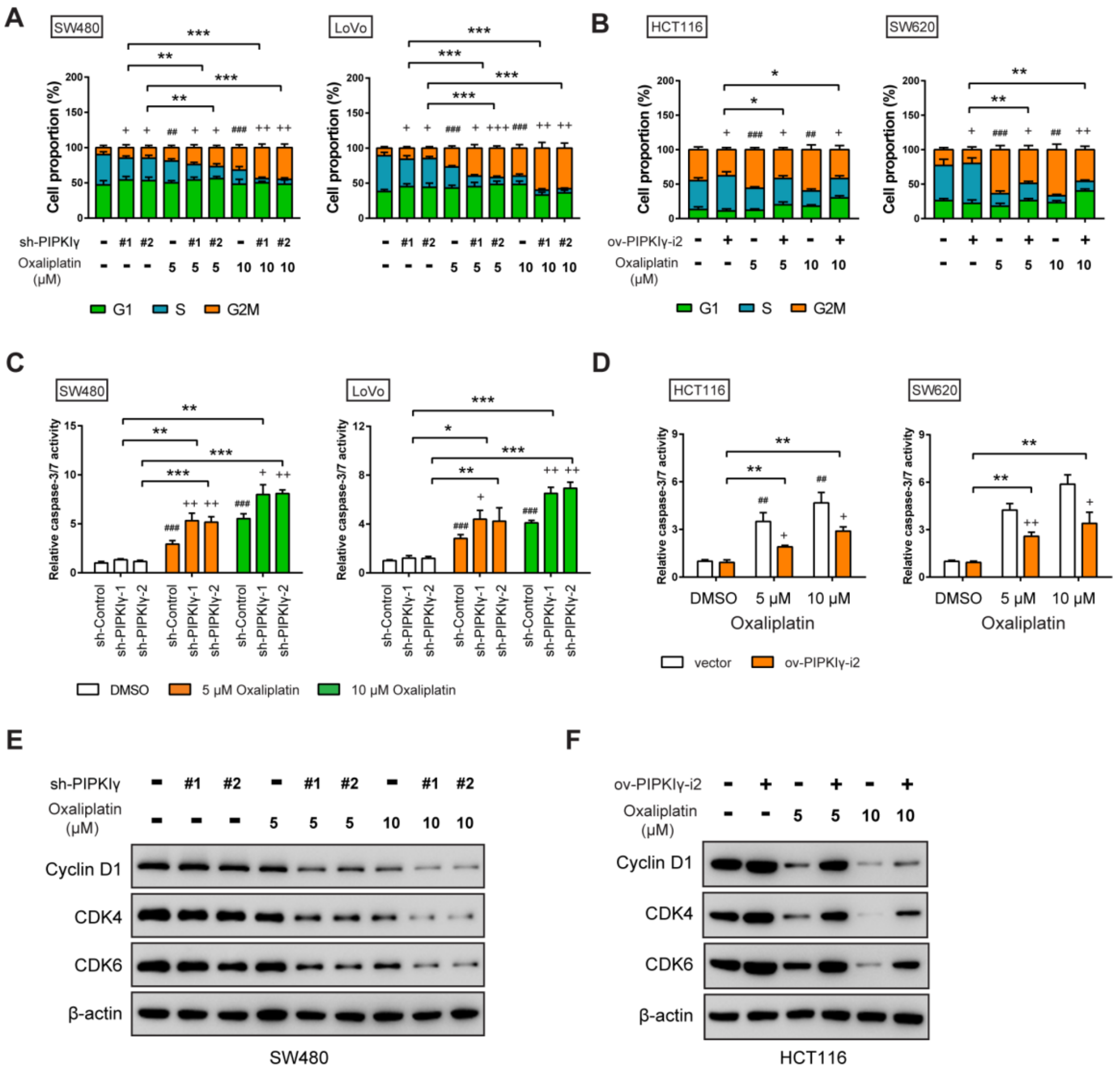

Figure. S1 PIPKIγ is involved in oxaliplatin resistance of CRC. (**A**) The effects of oxaliplatin treatment on sh-Ctrl and sh-PIPKIγ SW480 and LoVo cell cycle distribution. (**B**) The effects of oxaliplatin treatment on ov-vector and ov-PIPKIγ-i2 HCT116 and SW620 cell cycle distribution. (**C**) The effects of oxaliplatin treatment on sh-Ctrl and sh-PIPKIγ SW480 and LoVo cell apoptosis were measured by Caspase-3/7 activity assay. (**D**) The effects of oxaliplatin treatment on ov-vector and ov-PIPKIγ-i2 HCT116 and SW620 cell apoptosis were measured by Caspase-3/7 activity assay. (**E**) Western blotting analysis of Cyclin D1, CDK4, and CDK6 in sh-Ctrl and sh-PIPKIγ SW480 cells in the presence of different concentrations of oxaliplatin

treatment. (**F**) Western blotting analysis of Cyclin D1, CDK4, and CDK6 in ov-vector and ov-PIPKIγ-i2 HCT116 cells in the presence of different concentrations of oxaliplatin treatment. + indicates comparisons between sh-PIPKIγ and sh-Ctrl or comparisons between ov-vector and ov-PIPKIγ-i2, $^{+}P < 0.05$, $^{++}P < 0.01$, $^{+++}P < 0.001$; # represents comparison between DMSO and oxaliplatin treatment, $^{\#\#}P < 0.01$, $^{\#\#\#}P < 0.001$; asterisks represent indicated comparisons, *P < 0.05, **P < 0.01, ***P < 0.001. P values are derived from the ANOVA followed by post hoc Tukey's multiple comparison test.

Supplementary Figure 2

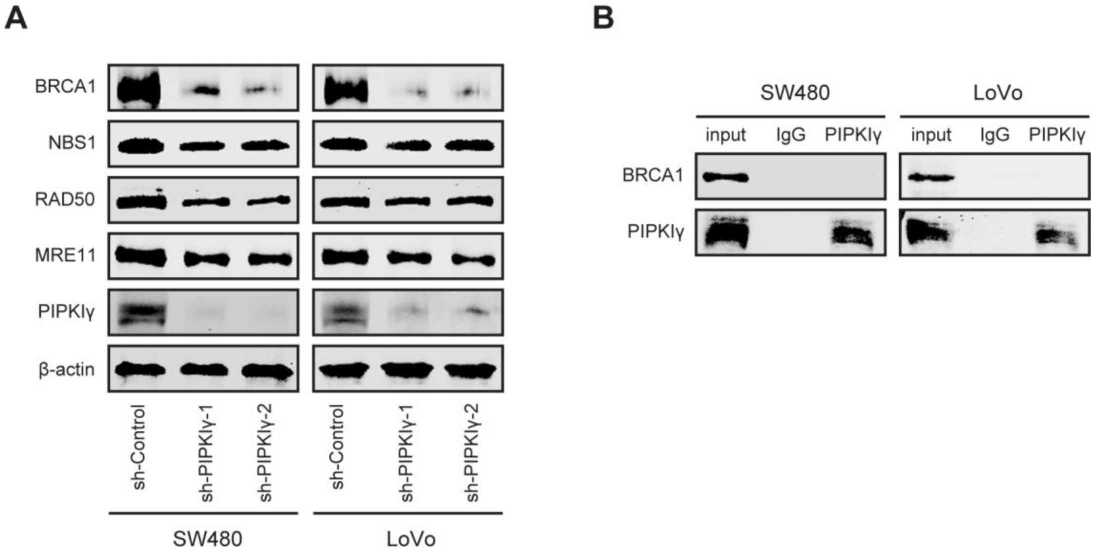

Figure. S2 The effects of PIPKIγ knockdown on the expression of DNA damage repair proteins in CRC cells. (**A**) Western blotting showed that after PIPKIγ was silenced, the expression of DNA damage repair-related proteins BRCA1, NBS1, RAD50, MRE11, and BRCA1 were decreased. (**B**) Co-IP experiment showed that PIPKIγ had no direct interaction with BRCA1 protein.

Supplementary Figure 3

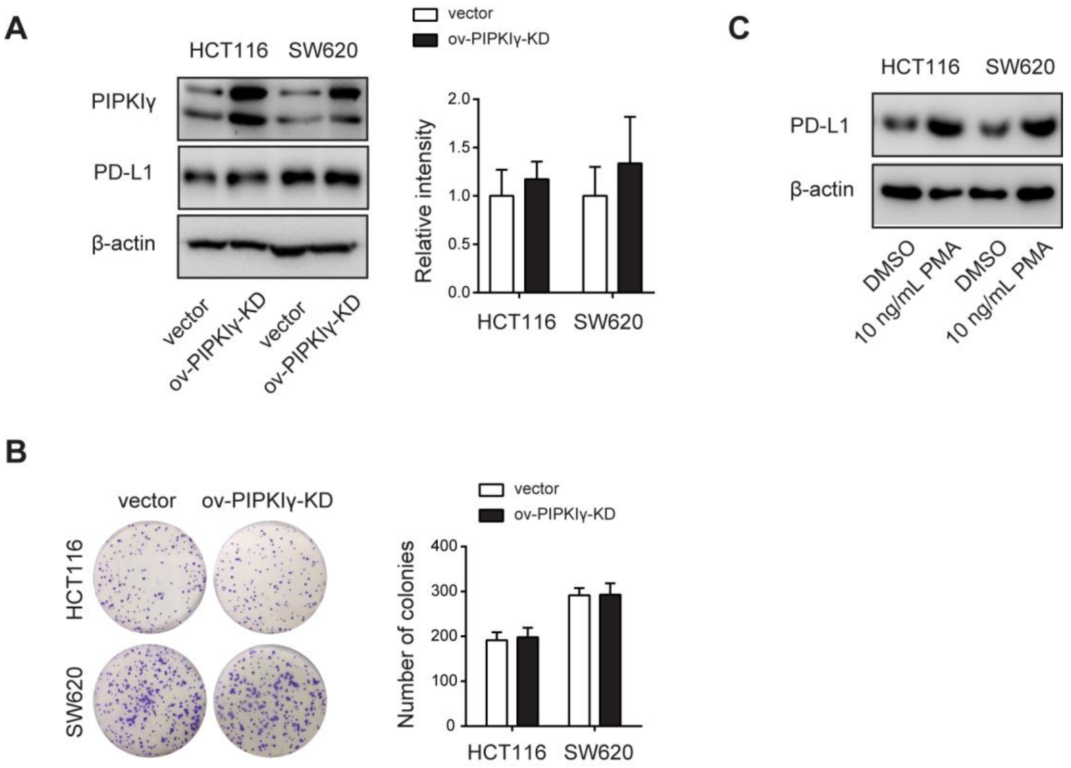

Figure. S3 PIPKIγ activity is required for inducing PD-L1 expression and promoting CRC proliferation. (**A**) Western blotting showed the overexpressed kinase-dead PIPKIγ (PIPKIγ-KD) and PD-L1 expression in HCT116 and SW620 cells. (**B**) The effects of PIPKIγ-KD overexpresion on HCT116 and SW620 cell proliferation were determined by plate colony formation. (**C**) Western blotting showed PD-L1 expression in HCT116 and SW620 cells upon stimulation with 10 ng/mL phorbol 12-myristate 13-acetate (PMA, PKC activator).

Supplementary Figure 4

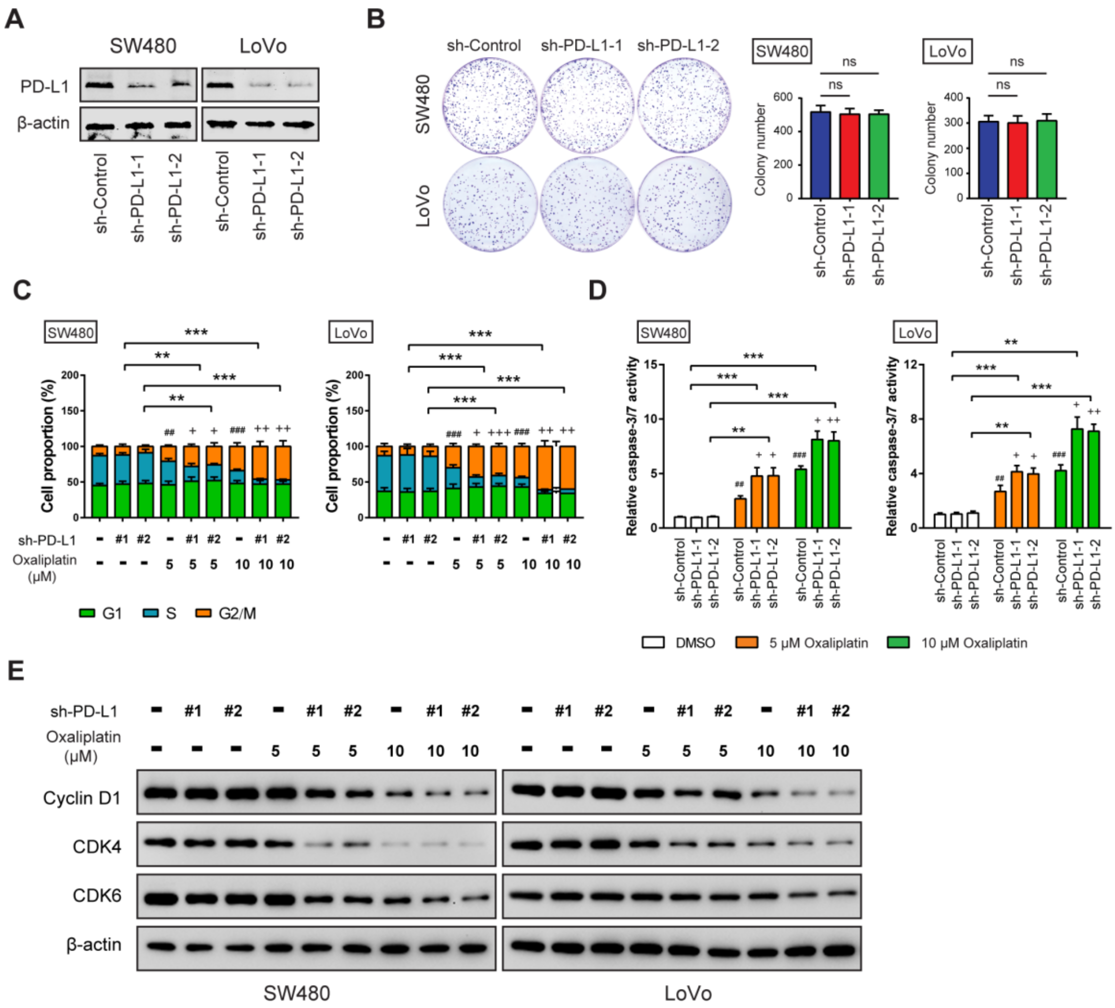

Figure. S4 The impacts of PD-L1 knockdown on CRC cell response to oxaliplatin treatment. (**A**) Western blotting showed the knockdown efficiency of PD-L1 in SW480 and LoVo cells. (**B**) The effects of PD-L1 knockdown on SW480 and LoVo cell proliferation were determined by plate colony formation; ns: not significant. (**C**) The effects of oxaliplatin treatment on sh-Ctrl and sh-PD-L1 SW480 and LoVo cell cycle distribution. (**D**) The effects of oxaliplatin treatment on sh-Ctrl and sh-PD-L1 SW480 and LoVo cell apoptosis were measured by Caspase-3/7 activity assay. (**E**) Western blotting analysis of Cyclin D1, CDK4, and CDK6 in sh-Ctrl and sh-PIPKIγ SW480 and LoVo cells in the presence of different concentrations of oxaliplatin treatment. + indicates comparison between sh-PD-L1 and sh-Ctrl, $^{+}P < 0.05$, $^{++}P < 0.01$, $^{+++}P < 0.001$; # represents comparison between DMSO and oxaliplatin treatment, $^{\#\#}P < 0.01$, $^{\#\#\#}P < 0.001$; asterisks represent indicated comparisons, $^{**}P < 0.01$, $^{***}P < 0.001$. P values are derived from the ANOVA followed by post hoc Tukey's multiple comparison test.

Supplementary Figure 5

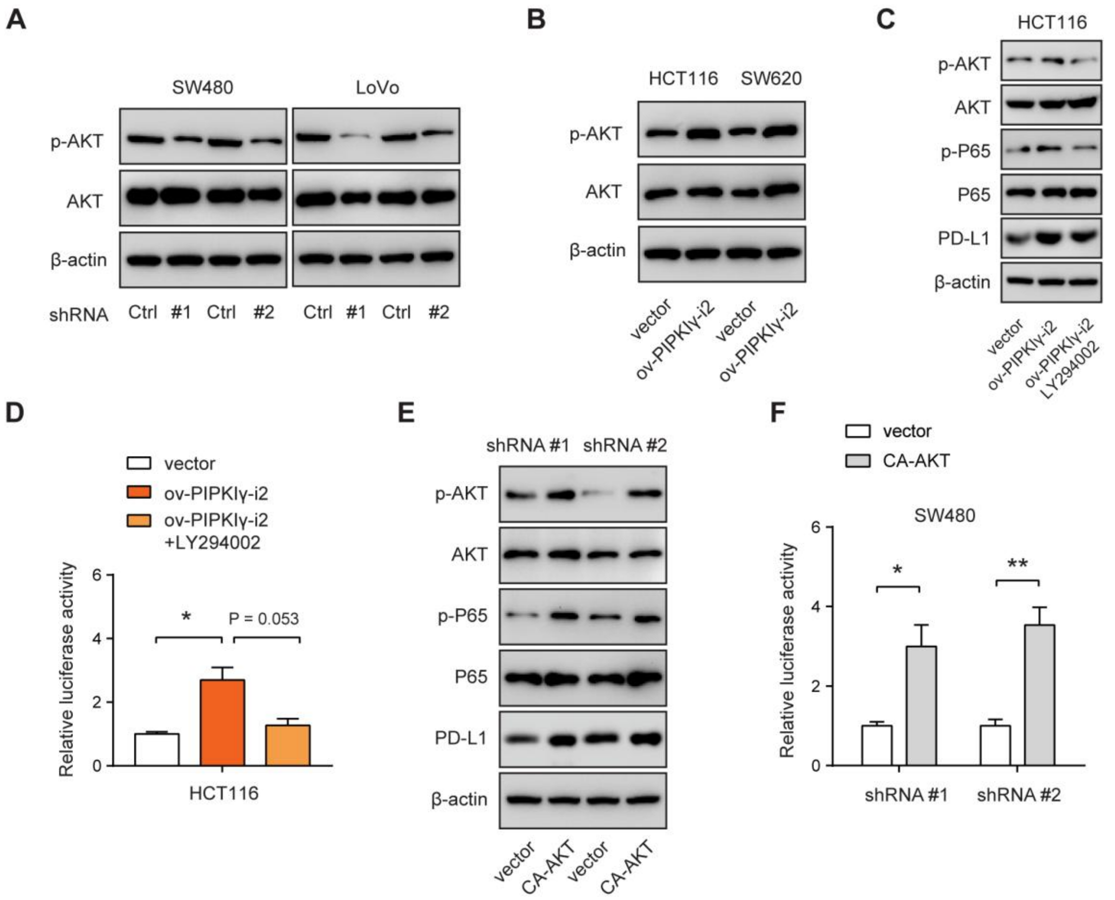

Figure. S5 AKT-dependent mechanism for PIPKIγ-induced of NF-kB activation. (**A**) The effects of PIPKIγ knockdown on the activation of AKT signaling pathway in SW480 and LoVo cells were analyzed by Western blotting. (**B**) The effects of PIPKIγ overexpression on the activation of AKT signaling pathway in HCT116 and SW620 cells were analyzed by Western blotting. (**C**) The effects of PIPKIγ overexpression on the activation of AKT signaling pathway was measured in the presence of LY294002 treatment. (**D**) The effects of PIPKIγ overexpression and LY294002 treatment on the transcriptional activity of NF-κB in HCT116 cells were determined by luciferase reporter assay. (**E**) Western blotting analysis of the effect of CA-AKT on NF-κB and AKT signaling pathways and PD-L1 expression in SW480 cells. (**F**) The effects of CA-AKT on the NF-κB transcriptional activity in sh-PIPKIγ SW480 cells were determined by luciferase reporter assay. *P < 0.05; **P < 0.01. P values are derived from the ANOVA followed by post hoc Tukey's multiple comparison test (D) or the Student paired t-test (F).

Table S1. Oxaliplatin-resistance genes in CRC

Gene name	logFC	P.Value
CLCA1	-2.161114	0.01092587
DEFA5	-1.989928	0.004355384
DEFA6	-1.959123	0.003499632
HEPACAM2	-1.825859	0.001175673
ITLN1	-1.735658	0.027533234
SPINK4	-1.668864	0.019051797
OLFM4	-1.61546	0.021160334
ATOH1	-1.614201	0.000472378
DMBT1	-1.548739	0.018940615
ADH6	-1.506447	5.48E-05
HULC	-1.498108	0.032823014
B3GNT6	-1.384889	0.024274703
TFF1	-1.350707	0.001777665
MPV17L	-1.284283	0.005088923
BTNL8	-1.272524	0.001356997
UGT2B7	-1.243336	0.000706197
BCAS1	-1.23781	0.000171198
GIF	-1.236522	0.00273013
SERPINA6	-1.200666	0.010652147
RETNLB	-1.147468	0.037943001
GP2	-1.139791	0.023272516
MMP3	-1.132727	0.011793611
UGT2A3	-1.12936	0.03487119
DNASE1L3	-1.126954	0.005194625
SERPINA7	-1.118738	0.008775243
UBD	-1.114225	0.001039444
MMP10	-1.113563	0.005774099
BMP5	-1.105084	0.002686128
SLC18A1	-1.103803	0.016722336
FCGBP	-1.10376	0.049681289
CHST5	-1.102272	0.030466696
IDO1	-1.092085	0.008884381
MMP1	-1.087006	0.010464265
IGJ	-1.082998	0.022398257
AKR1B10	-1.071343	0.019049504
BMPER	-1.064884	0.002781295
INSC	-1.04061	0.000399268
C9orf71	-1.016737	0.028862707
OTC	-1.014571	0.035149951
PALM3	1.0054376	0.003778327
COL11A2	1.026482	0.000243212
NMU	1.0269224	0.034076216

TREML2	1.034112	0.00399341
TACSTD2	1.0400472	0.019762077
CST6	1.0485672	0.004299364
ZNF750	1.051312	0.002203568
EYA1	1.0541151	0.021378366
HOXA2	1.0572291	0.000138306
PEG10	1.0683002	0.011523863
DLX3	1.0694159	0.022939725
PTPRN	1.0700265	0.002421547
MAGEA2	1.0863982	0.03658436
NTSR1	1.1049782	0.014005426
GJB5	1.1124754	0.041274687
CACNG4	1.1161053	0.034579016
PCDHA4	1.1280834	0.010550366
PLAG1	1.164368	0.000112873
MAGEA12	1.1748404	0.018854794
GABRA3	1.1957201	0.001782785
GABRB2	1.1990674	0.004568262
SLC14A1	1.2184835	0.012924748
LOC613037	1.2263369	0.000909173
VGLL1	1.2275371	0.000260211
CKMT2	1.2301707	0.046869539
LEMD1	1.2345389	0.00314105
MSLN	1.2353461	0.007389189
SCEL	1.2676419	0.011188182
ALOX15	1.2761463	0.000476985
UCA1	1.2797788	0.005629735
FGF19	1.316359	0.00761565
ALPPL2	1.3184951	0.007442914
HOXB8	1.3231272	0.001184895
KRT6A	1.3301393	0.018405061
TRIM58	1.339309	0.002228268
H19	1.3769059	0.00187277
CD274	1.3845756	0.026762765
HEPHL1	1.3848881	0.002815277
EDAR	1.3962848	0.001255283
DCDC2	1.4122274	0.004048241
ALPP	1.4650183	0.00057931
NPSR1	1.4762989	0.017990801
IGFL1	1.4787268	0.000373089
GSTM1	1.481303	0.012689625
MAGEA3	1.4828519	0.014946781
SFTA2	1.5200546	0.000128369
TH	1.5430092	1.58E-06

IGF2BP1	1.558037	0.004337718
DSG3	1.5921268	0.003293237
MAGEA6	1.5966503	0.014832113
PRSS33	1.6128732	0.000934557
UPK2	1.6471222	4.63E-07
PIP5K1C	2.088808	0.000694763
C6orf15	2.1593845	2.13E-05
